# Humoral immune response to tick-borne encephalitis vaccination in allogeneic blood and marrow graft recipients

**DOI:** 10.1038/s41541-020-00215-1

**Published:** 2020-07-24

**Authors:** Nicole Harrison, Katharina Grabmeier-Pfistershammer, Alexandra Graf, Ilse Schwarzinger, Judith H. Aberle, Karin Stiasny, Hildegard Greinix, Werner Rabitsch, Peter Kalhs, Michael Ramharter, Heinz Burgmann, Christina Forstner

**Affiliations:** 1 Division of Infectious Diseases and Tropical Medicine, Department of Medicine I, Medical University of Vienna, Vienna, Austria; 2 Division of Clinical and Experimental Immunology, Institute of Immunology, Center for Pathophysiology, Infectiology and Immunology, Medical University of Vienna, Vienna, Austria; 3 Section of Medical Statistics, Center for Medical Statistics, Informatics and Intelligent Systems, Medical University of Vienna, Vienna, Austria; 4 Department of Laboratory Medicine, Medical University of Vienna, Vienna, Austria; 5 Center for Virology, Medical University of Vienna, Vienna, Austria; 6 Division of Hematology, Department of Internal Medicine, Medical University of Graz, Graz, Austria; 7 Bone Marrow Transplantation Unit, Department of Medicine I, Medical University of Vienna, Vienna, Austria; 8 Department of Tropical Medicine, Bernhard Nocht Institute for Tropical Medicine & I. Department of Medicine, University Medical Center Hamburg-Eppendorf, Hamburg, Germany; 9 Institute of Infectious Diseases and Infection Control, Jena University Hospital, Jena, Germany

**Keywords:** Haematological cancer, Viral infection, Inactivated vaccines

## Abstract

The aim of this prospective study was to characterize the humoral immune response to TBE vaccination after hematopoietic stem cell transplantation (HSCT). Nineteen adult patients 11–13 months after HSCT and 15 age-matched immunocompetent adults received up to three TBE vaccinations. Antibodies against TBE virus were measured by neutralization test (NT). As primary endpoint, the antibody response (NT titer of ≥10 and at least a twofold increase from baseline 4 weeks after second vaccination) was compared between patients and controls using Fisher exact test. Prior vaccination, 15 (79%) HSCT patients still had detectable neutralizing antibodies. At primary endpoint, the antibody response was significantly lower in patients than in controls (35% versus 93%; *p* < 0.001). The CD4+ cell count was a predictor for an antibody response in patients (*p* = 0.019). Interestingly, the majority of HSCT patients still had detectable antibodies prior vaccination. Following vaccination, antibody response in HSCT patients was associated with the CD4+ cell count.

## Introduction

Allogeneic hematopoietic stem cell transplant (HSCT) and marrow graft recipients experience an increased risk for infections due to delayed immune reconstitution, immunosuppressive therapy, and graft-versus-host disease (GvHD)^1,2^. Guidelines recommend complete re-immunization against vaccine-preventable diseases after HSCT assuming that protection attained prior to transplantation is lost or at least strongly reduced^3–6^. Considering that lymphocytes need several months before they are mature enough to produce an effective vaccine response, the right timing after HSCT is difficult to determine. Furthermore, the effects of GvHD and immunosuppressive treatment might delay the process of immune reconstitution and limit the effectiveness of vaccination^2^. Recent guidelines recommend starting with vaccination against influenza, pneumococcal infection, and *Haemophilus influenzae* type b as early as 3 months after HSCT irrespective of whether the patient has or has not developed GvHD^7^. While certain vaccines like the conjugate pneumococcal vaccine have been evaluated by several studies^8–10^, there exist no data on effectiveness of tick-borne encephalitis (TBE) vaccine in patients after HSCT^4,5^. In Central and Eastern Europe, TBE is the most common viral infectious disease transmitted by infected ticks. Without protection provided by active immunization, tick-borne encephalitis virus (TBEV) can cause severe injuries of the nervous system or even death^11,12^. Austria belongs to the most affected TBEV areas in Europe^13^ and TBE emerged in new regions in Europe in 2018^14^. There are two inactivated whole-virus vaccines, based on Central European strains of the TBEV, available for adults (FSME-Immun®, Encepur-Adults®) and for children (FSME-Immun Junior®, Encepur-Children®), which are also effective against the Siberian and the Far Eastern subtypes of the virus^15,16^. Both vaccines have shown good efficacy and long-term persistence of antibodies in healthy children and adults^17^, although an impaired response was reported in persons aged >50 years^18^, in heart transplant recipients^19^, and patients with HIV^20,21^ and rheumatoid arthritis^22^. Therefore, a reduced immune response to the TBE vaccine in patients after HSCT has to be expected.

The aims of this prospective single-center pilot study were to characterize the immune response to vaccination against TBE in allogeneic HSCT recipients 1 year after transplantation compared to healthy controls and to evaluate the impact of age, gender, immune reconstitution, presence of GvHD, and other relevant factors on the vaccine response in HSCT patients.

## Results

### General characteristics of study population

**Fig. 1:**
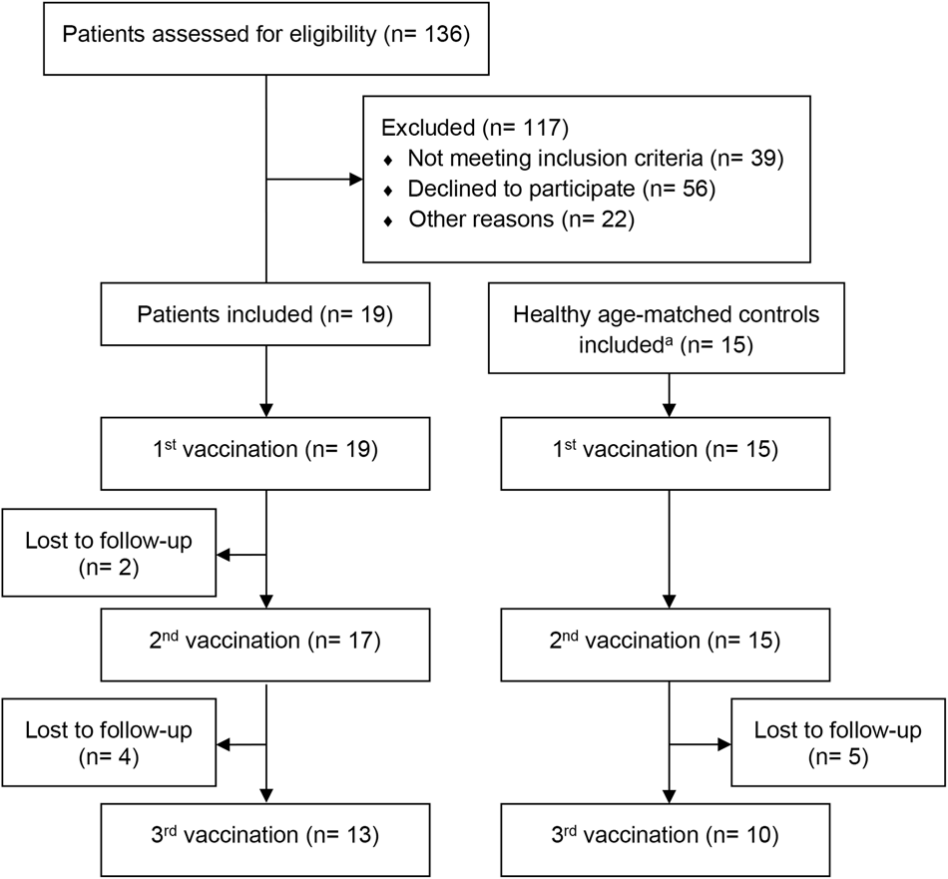
Flow chart of the study population depicting screening, entrollment and loss to follow-up. In the flow chart the number of patients who were screened for this study and the reasons for exclusion are documented. Number of patients and healthy controls who received one, two or all three vaccinations and the number of patients/healthy controls who were lost to follow-up at each step of the study are depicted. ^a^The total number of individuals screened for eligibility was not recorded.

From July 2014 to January 2018, 19 patients and 15 healthy controls were included in this study. Overall, 136 patients after allogeneic HSCT were screened and the recruitment rate was 14% (Fig. 1). Patients were included 11–13 months (median 12.5 months, range 11–13.5) after HSCT. Two HSCT patients prematurely terminated the study due to mild general symptoms after first vaccination (cold-like symptoms in one patient and new appearance of skin GvHD in the other patient, these symptoms occurred 4 weeks after first vaccination in both patients) and were therefore not included in the final analysis. Patients’ characteristics are depicted in Table 1. All patients except one and all sibling donors previously received a complete basic vaccination schedule and at least one TBE booster vaccination before HSCT. The time of the last booster vaccination of individual patients and sibling donors before HSCT/donation is shown in Table 2.

**Table 1:** Characteristics of patients (*n* = 17) included in the final analysis and pretransplant and posttransplant details of their allogeneic hematopoietic stem cell transplantation (HSCT).

Characteristics of HSCT recipients	Total number	%
Underlying disease		
Acute myeloid leukemia (AML)	12	70.6
Acute lymphoblastic leukemia	2	11.8
Lymphoma	2	11.8
Aplastic anemia	1	5.9
Conditioning regimen		
Myeloablative	8	47.1
Reduced intensity	9	52.9
Graft		
Peripheral blood stem cells	16	94.1
Bone marrow	1	5.9
Donor		
Related donor	9	52.9
Unrelated donor	8	47.1
HLA match		
Match	16	94.1
Mismatch	1	5.9
EBMT score^32^		
Score ≤2 (low risk)	6	35.3
Score >2 (high risk)	11	64.7
Previous HSCT		
First HSCT	14	82.4
Previous allogeneic HSCT	1	5.9
Previous autologous HSCT	2	11.8
Immunosuppression^a^		
No immunosuppressive therapy	6	35.3
Immunosuppressive therapy^b^	11	64.7
Corticosteroids	4	23.5
Cyclosporine	7	41.2
Tacrolimus	1	5.9
Sirolimus	1	5.9
Severity of prior acute GvHD		
None	13	76.5
I–II	3	17.6
III–IV	1	5.9
Chronic GvHD		
None	5	29.4
I–II (mild to moderate)	12	70.6
III–IV (severe)	0	0
Affected organs^b^		
Skin	4	23.5
Mucosa	4	23.5
Liver	9	52.9
Eyes	2	11.8
Comorbidities prior vaccination		
Cardiovascular disease	4	23.5
Diabetes mellitus	2	11.8
Thrombosis or embolism	3	17.6

^a^Immunosuppressive therapy at the time of first vaccination.

^b^More than one possible.

*EBMT* European Society of Blood and Marrow Transplantation, *GvHD* graft-versus-host disease, *HLA* human leukocyte antigen.

**Table 2:** Overview of TBE immunizations in sibling donors and recipients before allogeneic hematopoietic stem cell transplantation (HSCT).

HSCT patients						Sibling donors			
Patient	Complete basic TBE immunization pre-HSCT	Time of last TBE booster vaccination pre-HSCT	NT titer pre-HSCT	NT titer post-HSCT	Decline of NT titer^a^	Sibling donor	TBE immunization pre-donation	Time of last TBE booster vaccination pre-donation	NT titer pre-donation
	Yes/no	Months			%	Yes/no	Yes/no	Months	
1	Yes	Unknown	40	10	75.0	Yes	Yes	Unknown	67
2	Yes	51	453	160	64.7	Yes	Yes	13	160
3	Yes	92	135	95	29.6	Yes	Yes	71	40
4	Yes	Unknown	113	40	64.6	No	–	–	–
5	Yes	129	67	48	28.4	Yes	Yes	Unknown	67
6	Yes	194	113	113	0	No	–	–	–
7	Yes	24	40	6	85.0	No	–	–	–
8	Yes	58	>640	538	>15.9	Yes	Yes	38	160
9	Yes	11	40	12	70.0	No	–	–	–
10	Yes	Unknown	135	40	70.4	No	–	–	–
11	Yes	54	160	40	75.0	No	–	–	–
12	Yes	177	>640	10	>98.4	Yes	Yes	Unknown	Unknown
13	Yes	20	>640	10	>98.4	Yes	Yes	60	538
14	No	–	10	<5	>50.0	No	–	–	–
15	Yes	49	>640	80	>87.5	No	–	–	–
16	Yes	40	160	135	15.6	Yes	Yes	54	190
17	Yes	186	67	<5	>92.5	Yes	Yes	25	190

^a^Between pre-HSCT and post-HSCT prior TBE (re-)vaccination.

### Assessment of antibody response after second vaccination by neutralization assay and enzyme-linked immunosorbent assay (ELISA)

At the primary endpoint, Fisher exact test showed a significant difference between HSCT patients and healthy controls in the antibody response measured by neutralization test (NT) 4 weeks after second vaccination (*p* < 0.001). A significantly higher proportion of individuals of the control group achieved antibody response by NT test (14/15, 93.3%) as compared to the patient group (6/17, 35.3%). To investigate this in more detail, a logistic regression model was performed for antibody response accounting for group (patients versus controls), age, body mass index (BMI), and gender (female versus male). No significant impact of age, BMI, or gender on antibody response was found, but belonging to the patient group remained significant in the multivariable model (adjusted odds ratio 0.025, 95% confidence interval (CI) 0.001–0.44, *p* = 0.012). In the patient group, 5 of the 6 patients, who achieved antibody response, had preexisting antibodies to TBE, while in the control group none of the participants had preexisting antibodies.

A similar result was found for the ELISA. Fisher exact test showed a significantly larger antibody response rate measured by ELISA for the control group (14/15, 93.3%) as compared to the patient group (9/17, 52.9%, *p* = 0.018). Comparing ELISA and NT results, 5 of the 32 (15.6%) study participants showed different results in both tests (4 patients with negative NT tests were positive by ELISA and 1 patient with positive NT test was negative by ELISA) with Cohen’s Kappa of 0.65 (95% CI 0.36–0.94). In the control group, all participants showed complete agreement in both test results (15 of 15, Cohen’s Kappa 1.0).

### Assessment of geometric mean titers (GMTs) and geometric mean fold rises at different time points by neutralization assay

At baseline before first vaccination, patients’ geometric mean NT titer (31.8 95% CI 15.2–66.6) was significantly higher (Wilcoxon test *p* < 0.001) than that of healthy controls, whose titer was under the detection limit (NT titer <5). Considering that an NT titer of ≥10 is considered protective, 15 of the 19 (79%) enrolled patients and 14 of the 17 (82%) patients included in the final analysis had protective NT titers at baseline; 1 developed an NT titer ≥10 after 2 vaccinations and 1 after 3 vaccinations. The third patient was lost to follow-up after two vaccinations. In total, four patients were lost to follow-up after two vaccinations. Therefore, 88% (15/17) of patients receiving 2 vaccinations and 100% (13/13) of patients receiving 3 vaccinations showed protective titers.

**Fig. 2:**
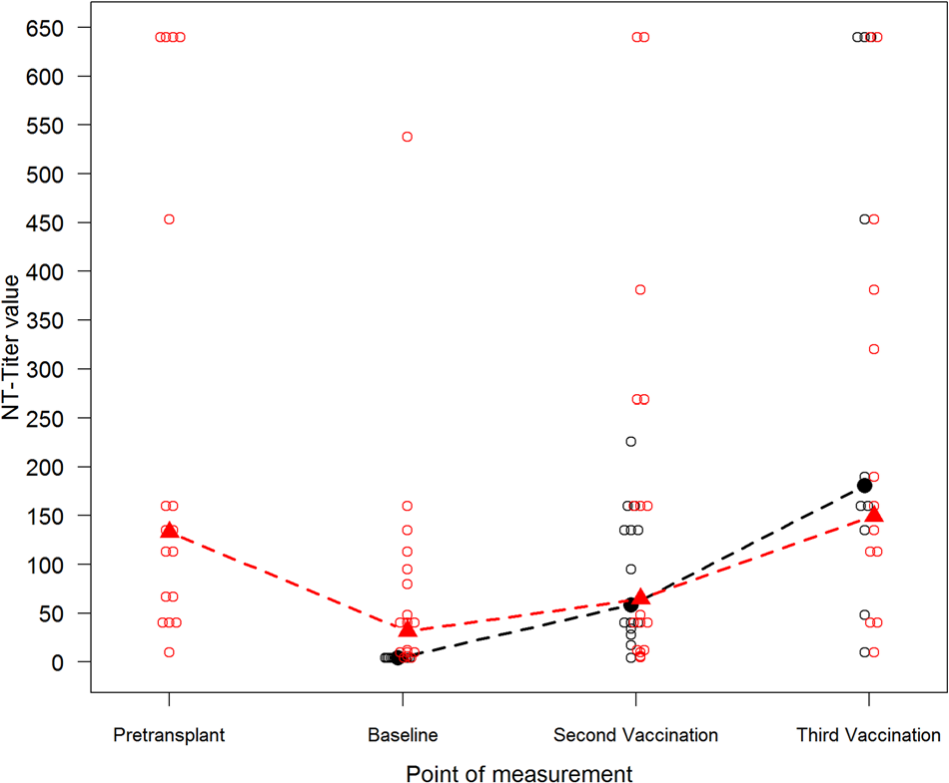
Assessment of geometric mean and individual NT titers. Geometric mean NT titers (filled triangles and circles) and individual titers (blank circles) of patients (red) versus controls (black) at pretransplant, at baseline 1 year after HSCT, 4 weeks after second vaccination, and 4 weeks after third vaccination.

The patients’ geometric mean NT titer was 64.7 after second (95% CI 28.1–149.4) and 149.7 after third vaccination (95% CI 71.5–313.4). The controls’ geometric mean NT titer was 58.5 after second (95% CI 32.2–106.1) and 180.9 after third vaccination (95% CI 69.9–467.9) (see Fig. 2). In HSCT patients, Pearson’s correlation coefficient indicated a moderate positive linear correlation between NT titers at baseline and NT titers 4 weeks after second vaccination (Pearson’s *r* = 0.638, *p* = 0.006).

**Fig. 3:**
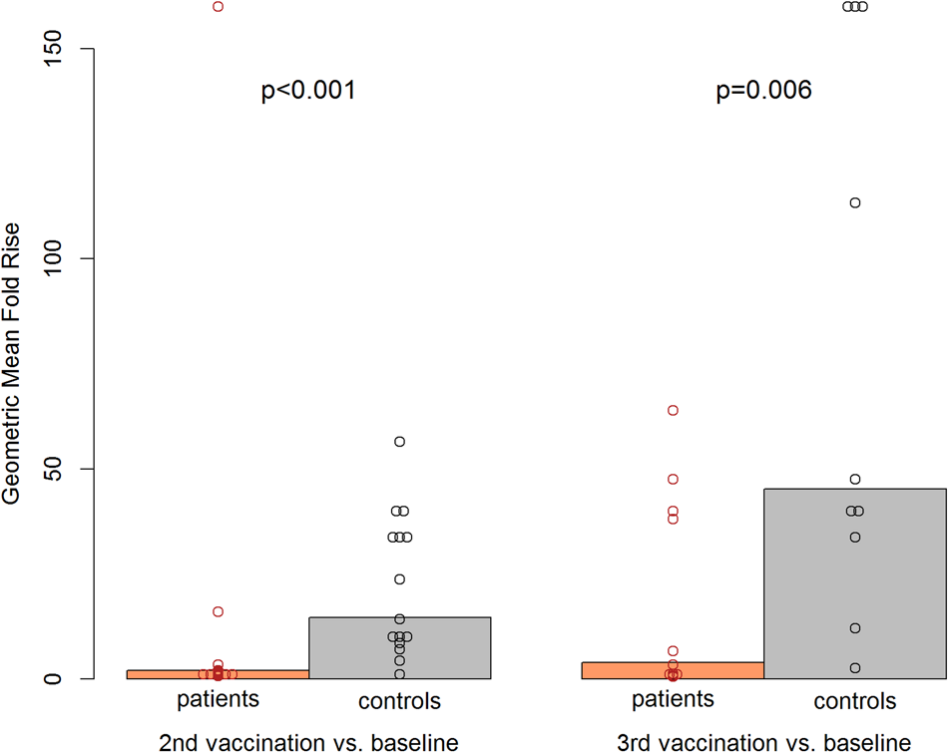
Assessment of geometric mean fold and individual NT titer rises. Geometric mean fold rises of NT titers (bars) and individual rises (circles) from baseline to 4 weeks after second vaccination and from baseline to 4 weeks after third vaccination comparing patients (red) with healthy controls (black) with corresponding *p* values.

When comparing the geometric mean fold rises between time after second vaccination and baseline, a significant difference was observed between patients and controls. The geometric mean fold rise was 14.6 (95% CI 8.1–26.5) in the control group versus 2.0 (95% CI 1.0–4.1) in the patient group (Wilcoxon test *p* < 0.001) (Fig. 3). A significant difference (*p* = 0.006) was also found from baseline to time after third vaccination with a geometric mean fold rise of 45.2 (95% CI 17.5–117.0) for controls compared to 3.9 (95% CI 1.3–11.9) for patients (see Fig. 3).

The patients’ geometric mean NT titer pretransplant (median 25 days before HSCT, range 7–88 days before HSCT) was 133.2 (95% CI 71.4–248.2) and decreased to 31.8 (95% CI 15.2–66.6) 1 year after transplantation at baseline, corresponding to a geometric mean fold change of 0.24 (95% CI 0.12–0.45). Decline of NT titer between pre-HSCT and post-HSCT prior TBE re-vaccination of individual patients is shown in Table 2. All tested sibling donors had similar protective titers (8/9, 1 missing data) before stem cell donation with a geometric mean NT titer of 131.5 (95% CI 66.5–260.0). Samples of unrelated donors were not available.

### Logistic regression of predictors for antibody response after second vaccination

Next, predictors for antibody response after second vaccination were calculated for the patient group. In univariate analysis (Table 3), T cell reconstitution at baseline (meaning normal CD4+ and CD8+ cell counts) (*p* = 0.033) and higher numbers of CD4+ T cells (*p* = 0.019) were significantly associated with an antibody response. Although not statistically significant, female patients were more likely to experience an antibody response (66.7%) than male patients (18.2%) and patients with related donors (55.6%) showed more responders than patients with unrelated donors (12.5%). In the multivariate model, only the absolute CD4+ cell count remained significantly associated with an antibody response (*p* = 0.019). The area under the receiver operating characteristic curve of CD4+ cell count at baseline to predict antibody response was 0.864 (95% CI 0.685–1.0). For a cut-off point of 390 CD4+ cells/µl, the sensitivity was 83.3% and the specificity was 81.8% to select patients with antibody response after second vaccination. In the group of patients with CD4+ cell count <390/µl (*n* = 10), there was 1 vaccination responder, and in the group of patients with CD4+ cell count ≥390/µl (*n* = 7), there were 5 vaccination responders (83.3%). Nevertheless, no significant difference was found (*p* > 0.1) comparing the percentage of memory (CD4+CD45RO+) and naive (CD4+CD45RA+) CD4+ cells between vaccination responders and non-responders. In addition, total B cell counts and the proportion of B cell subsets including immature/transitional, class- and non-class-switched memory cells, and plasmablasts did no significantly distinguish patients responding and non-responding to TBE vaccination (*p* > 0.1). However, the majority of patients exhibited subnormal numbers of memory B cells (median 26.7 cells/µl), with only 11.8% reaching normal levels of class-switched memory B cells and 52.9% of non-class-switched memory B cells.

**Table 3:** Univariate logistic regression model for predictors for antibody response after second vaccination (patients only).

Variable	Patients with antibody response^a^ (*n* = 6)	Patients without antibody response^a^ (*n* = 11)	Odds ratio (95% confidence interval)	*p* Value
Age in years, median (IQR)	27 (25–38.5)	43 (26–55)	0.96 (0.88–1.03)	0.250
BMI, median (IQR)	23.3 (19.4–31.5)	27.8 (24–31.5)	0.94 (0.76–1.09)	0.417
Female gender	4 (66.7%)	2 (18.2%)	6.84 (0.94–67.18)	0.058
AML	5 (83.3%)	7 (63.3%)	2.20 (0.29–26.79)	0.459
Myeloablative conditioning regimen	4 (66.7%)	4 (36.4%)	3.00 (0.45–24.16)	0.257
Related donor	5 (83.3%)	4 (36.4%)	6.11 (0.83–75.72)	0.077
Prior acute GvHD	1 (16.7%)	3 (27.3%)	0.66 (0.05–5.49)	0.709
Chronic GvHD	4 (66.7%)	8 (72.7%)	0.74 (0.10–5.94)	0.768
Any immunosuppressive medication	4 (66.7%)	7 (63.6%)	1.08 (0.16–8.42)	0.938
EBMT score >2	3 (50%)	8 (72.7%)	0.41 (0.05–2.86)	0.366
T cell count/µl, median (IQR)	1220 (1144–1513)	766 (576–1115)	1.00 (1.00–1.00)	0.196
T cell reconstitution^b^	5 (83.3%)	3 (27.3%)	8.90 (1.18–115.07)	0.033
CD4+ cell count/µl, median (IQR)	456 (380–544)	315 (257–387)	1.01 (1.00–1.03)	0.019
CD4+CD45RO+ cells in %, median (IQR)	52.9 (47.2–71)	64.5 (45.6–73.7)	0.99 (0.94–1.05)	0.755
CD4+CD45RA+ cells in %, median (IQR)	26.5 (11.9–33.9)	17.8 (5.8–26.2)	1.03 (0.95–1.12)	0.474
CD8+ cell count/µl, median (IQR)	603 (476–1141)	340 (267–758)	1.00 (1.00–1.00)	0.614
CD4/CD8 ratio, median (IQR)	0.76 (0.33–1.15)	0.96 (0.42–1.16)	0.67 (0.08–4.59)	0.679
B cell count/µl, median (IQR)	358 (145–454)	380 (129–592)	0.999 (1.00–1.00)	0.584
Memory B cells in %, median (IQR)	10.7 (6.65–15.43)	5.9 (4.3–14.55)	1.10 (0.89–1.13)	0.866
IgG level in mg/dl, median (IQR)	913 (739–1133)	843 (557–1200)	1.00 (1.00–1.00)	0.569
IgA level in mg/dl, median (IQR)	115 (55–251)	88 (56–161)	1.01 (0.99–1.02)	0.323
IgM level in mg/dl, median (IQR)	72 (45–123)	97 (68–124)	1.00 (0.98–1.00)	0.459
NT titer pretransplant patient, median (IQR)	90 (60.3–280)	135 (40–640)	1.00 (0.99–1.00)	0.518
NT titer pretransplant donor, median (IQR)	160 (67–190)	160 (40–160)	1.00 (0.99–1.00)	0.380
NT titer baseline patient, median (IQR)	80.5 (19.5–129.5)	40 (10–60)	1.00 (0.99–1.02)	0.192

*AML* acute myeloid leukemia, *CD4+CD45RO+ cells* memory CD4+ cells, *CD4+CD45RA+ cells* naive CD4+ cells, *EBMT* European Group for Blood and Marrow Transplantation, *GvHD* graft-versus-host disease, *NT* neutralization titer.

^a^Antibody response was defined by an NT titer of ≥10 and at least a twofold increase of titer from baseline (or titer above the highest level of measurement).

^b^T cell reconstitution = normal CD4+ and CD8+ cell counts.

### Safety data

Overall, 52 adverse events (AEs) were observed during the study period. Most common was local pressure pain at the injection site (*n* = 20). The number of AEs was higher in the patient than in the control group, patients having a median number of 2 AEs/patient (range 0–9) and controls having a median number of 1 AE/controls (range 0–3, Wilcoxon test *p* = 0.02). In total, 42 AEs were found in the patient and 10 in the control group. Four serious AEs were reported in the patient group, all of which were not considered related to the vaccine (cholelithiasis, hematometra, influenza, relapse of leukemia). In two patients, increase of the severity of chronic GvHD was recorded after vaccination coinciding with reduction of systemic immunosuppression (mild skin rash in one patient with cutaneous GvHD 4 weeks after first vaccination and reduction of rapamycin from 2 to 1 mg, and increase of mucosal lesions from mild-to-moderate severity in one patient with oral mucosal GvHD 1 week after second vaccination and reduction of cyclosporine from 200 to 150 mg daily).

## Discussion

Although recent guidelines suggest re-vaccination against TBE after allogeneic HSCT in endemic areas^5^, the evidence for this recommendation is based on studies with other immunocompromised patient cohorts, more precisely with heart transplant recipients^19^. Therefore, no data existed regarding the optimal time after HSCT when TBE vaccination can be expected to induce a sufficient immune response^4^. Hence, this study assessed the humoral immune response to TBE vaccination in patients after HSCT living in an endemic region for TBEV. In addition, most HSCT patients (70%) in this study have been suffering from mild-to-moderate chronic GvHD. The main findings of our study were: first, at baseline, 11–13 months after HSCT, the majority of patients (79%) had decreased but still detectable antibodies (NT titer ≥10), considered to be protective at least in immunocompetent patients. Second, at primary endpoint antibody response to FSME-Immun® was significantly reduced in HSCT patients compared to healthy controls, but all HSCT patients achieved protective NT titers after completing basic immunization with three vaccinations. Third, the CD4+ cell count prior to vaccination was identified as a significant predictor for an antibody response in HSCT patients.

Current guidelines recommend starting re-vaccination against TBE in endemic areas 6–12 months after HSCT^5,23^. Interestingly, 1 year after allogeneic HSCT the majority of patients (79%) still exhibited neutralizing antibodies against TBEV. Although other studies have described that antibodies can persist after HSCT, the number of patients with detectable antibodies in our study was much higher than expected. However, as a pre-vaccination NT titer ≥10 was not an exclusion criterion of this study, also those patients with detectable NT titers received re-vaccination. Even the recently published recommendation of the Ständige Impfkommission (STIKO) May 2020 does not routinely recommend to determine antibody titers prior TBE re-vaccination in patients after allogeneic HSCT^24^. At least, we detected a strong decline of NT titer from before HSCT to 1 year after HSCT in most patients, and it can be assumed that antibody titers will decline further, and therefore even patients with protective titers after HSCT might benefit from re-vaccination.

Vaccination against TBE is well established in the Austrian population. Therefore, all patients in this study except one, as well as all sibling donors, were vaccinated before transplantation. Several studies provide evidence that immunity against vaccine-preventable diseases is transferred from the donor to the recipient^25^. However, there is also evidence that patients might retain their own immunity, e.g., patients with natural measles infection develop antibodies much longer after transplantation than patients who were vaccinated before HSCT^26,27^. In this study, patients with sibling donors were more likely to respond to TBE vaccination, although this was not statistically significant. Following these results, we retrospectively analyzed serum samples, which were collected from patients and sibling donors immediately before transplantation. All patients and sibling donors had detectable antibodies, but the strength of the antibody level was not a predictor for antibody response. However, considering the countries of origin of the unrelated donors (1 USA, 2 Poland, 5 Germany), it appears possible that vaccination rates were much lower in the unrelated donor group. Vaccination against TBE is not generally recommended in these countries and compliance with vaccination is even low in TBE-risk areas of Germany^28^. Unfortunately, there were no serum samples available from the unrelated donors to test this hypothesis any further. The influence of donor immunity against vaccine-preventable disease is certainly an area that should be investigated further.

Similar to other patient groups with secondary immunodeficiency^19–22^, HSCT patients showed a reduced immune response to TBE vaccination, as only 35% of patients but 93% of controls exhibited an at least twofold increase of the NT titer 4 weeks after the second TBE vaccination. Seroconversion rates in healthy volunteers detected by NT were as high as reported previously^29,30^: >90% after second vaccination and 100% after third vaccination. Although preexisting antibodies against TBEV could possibly be an explanation for weaker responses in HSCT patients versus healthy controls who had never received a TBE vaccine before, we found a moderate positive correlation between NT titers at baseline and after second vaccination in HSCT patients. In addition, the only HSCT patient without previous TBE vaccination did not respond after second vaccination (NT titer <10 at baseline and after second vaccination), but achieved a protective antibody response (NT titer 190) after third vaccination. Comparing antibody response measured by ELISA and NT at the primary endpoint, all participants of the control group showed complete agreement in both test results (15 of 15, Cohen’s Kappa 1.0). Five of the 17 patients (29.4%) showed differing results in both tests with a Cohen’s Kappa of 0.42. Therefore, for assessment of TBE-specific humoral immunity in HSCT patients, the time-consuming and costly NT test remains the gold standard and should not be replaced by ELISA, whereas ELISA produced reliable results in healthy volunteers.

One limitation of this study might be the definition of response to vaccination. Considering patients after HSCT, their response to vaccination cannot be defined the same way as in healthy individuals. The concept of seroconversion is not applicable as the majority of patients had preexisting antibodies, and therefore other parameters had to be considered in order to decide whether a patient actually responds to vaccination. A twofold rise in NT titer was quite moderate and might have overestimated the number of responders. However, considering that most patients had antibodies before vaccination, a fourfold increase was considered too strict as definition for vaccination response by the authors. Only two patients showed a fourfold increase in titer (in addition, one patient had a titer at the highest level of measurement), meaning that only three patients would have fulfilled this definition of response.

A further limitation of the study is the small sample size in the patient and control groups. We observed a rather large effect for the primary hypothesis to compare antibody response between patients and controls, which led to the significant result (also in the multivariable regression analysis). However, for the secondary question of the study to search for possible predictors for vaccination response within the patient group, the power may be low and corresponding *p* values may be interpreted as tendencies only. Classical parameters such as presence of immunosuppressants, mild-to-moderate chronic GvHD, hypoglobulinemia, or increasing age did not show a significant influence on early antibody response after TBE vaccination, which may be due to the small sample size. In contrast, the important role of reconstitution of immune cells, especially of CD4+ T cells, is underlined by our finding that the CD4+ cell count showed a significant result for an appropriate response to vaccination. We were able to determine a cut-off that might select between serological responders and non-responders at primary endpoint (positive >390 CD4+ cells/µl). At least in the present study, we did not detect a significant correlation between the severity of immunosuppressive treatment and the CD4+ cell count, as the CD4+ cell count did not significantly differ between patients with immunosuppressive therapy and patients without (median CD4+ cell count 358/µl versus 362/µl, *p* = 0.96).

Surprisingly, the number of B lymphocytes or different subpopulations had no significant influence on vaccination response at all, although most patients had subnormal memory B cells. Based on the current results, it can only be concluded that CD4+ T cells play an important role in vaccine response against TBE in patients after HSCT. In addition, it might be possible to postpone re-vaccination against TBE in patients with low CD4+ cells <390/µl but still detectable antibodies against TBEV.

The number of AEs was higher in HSCT patients than in healthy controls. However, most AEs were minor and all serious AEs were not considered related to the vaccine. Notably, two patients experienced an increase of GvHD severity after vaccination. In both patients, the immunosuppressive medication was reduced at the time of vaccination, possibly causing the flare in GvHD.

This study experienced several limitations, especially due to the difficulty in recruitment of patients as the inclusion rate was lower than expected. As a single-center study with a study population from the Eastern part of Austria, the data applies only to patients in an endemic region with a high vaccination rate of related HSCT donors and high pretransplant vaccination rates among HSCT recipients. Considering the low number of patients, recommendations can be cautiously based on this data but should be confirmed by larger studies. Moreover, even though NT titer is the Food and Drug Administration-accepted primary endpoint of immunogenicity of flavivirus vaccines^31^, TBE antigen-specific lymphoproliferative immune response might be of particular interest and was not addressed by this paper.

Apart from these limitations, this study provides relevant data to guide further recommendations for TBE vaccination in patients after HSCT. In countries with high vaccination rates like Austria, a high percentage of patients with detectable antibodies after HSCT can be expected. Three vaccinations as recommended for healthy persons were sufficient to acquire protective titers in all our patients.

In conclusion, a high percentage of patients after HSCT had decreased but still detectable TBE-specific neutralizing antibody titers 1 year after transplantation. However, the immune response to vaccination was still hampered as reflected by significantly lower titer rises after two TBE vaccinations compared to age-matched healthy controls. Overall, patients who had received a full vaccination course achieved protective titers. A significant predictor for vaccine response found by this study was the CD4+ cell count, prompting further investigations into the role of cellular immune response after TBE vaccination.

## Methods

### Study population and design

In this prospective single-center open-label study, adult patients aged ≥18 years were screened 11–13 months after allogeneic HSCT at the Outpatient Clinic of the Bone Marrow Transplant Unit of the University Hospital of Vienna, Austria. Exclusion criteria were prior TBE vaccination after transplantation; severe GvHD requiring treatment with more than two immunosuppressive drugs or receiving >0.5 mg/kg prednisone daily as part of an immunosuppressive combination therapy; relapse of the underlying malignant disease; severe allergic reactions or anaphylaxis to vaccines in the past; febrile illness in the past 2 weeks; pregnancy or breastfeeding in female patients; and previous TBEV infection, dengue virus infection, or vaccination against yellow fever or Japanese encephalitis. Healthy controls had to be at least 18 years of age, clinically healthy without any immunosuppressive condition including any history of immune-mediated diseases, long-term use of corticosteroids, hemodialysis, chronic renal insufficiency, liver cirrhosis Child-Pugh class C, hemato-oncological malignant disease, solid organ transplant or HSCT and without prior TBE vaccination, and vaccination against yellow fever or Japanese encephalitis or any flavivirus infection in their medical history. For recruitment of healthy controls, posters were displayed at the Medical University of Vienna including the Austrian Students’ Union and non-native healthcare professionals were directly addressed at our University Hospital. Participation was voluntary and all participants signed a written informed consent before enrollment. The study protocol was approved by the Ethics Committee of the Medical University of Vienna (No. 830/2011) and by the Austrian Competent Authorities (Bundesamt für Sicherheit im Gesundheitswesen) represented by the Agency for Health and Food Safety (AGES PharmMed). This study was registered with clinicaltrials.gov (NCT01991067).

The patient population and healthy control group were age matched (median age of 31 years in the patient group (range 22–61) compared to a median age of 30 years (range 21–60) in the control group). All patients and healthy controls received up to three doses of FSME Immun® (each dose contains 2.4 µg of inactivated TBE virus strain Neudörfl) intramuscularly—first at baseline, second after 4 weeks, and third after 9–12 months. Participants were asked to record all AEs in a patient diary for 4 weeks after each vaccination. During the first visit, demographic and medical data including underlying malignant disease leading to HSCT, conditioning regimen, type and donor of the hematopoietic graft, European Society of Blood and Marrow Transplantation risk score^32^, intensity of immunosuppressive therapy, medical history including time of previous TBE vaccination before HSCT, and present status of chronic GvHD according to the National Institutes of Health criteria^33^ were recorded.

### Study endpoints

The primary endpoint of this study was the antibody response after TBE vaccination as measured by neutralization assay (NT) 4 weeks after the second vaccination. Antibody response was defined as a composite endpoint by a NT titer of ≥10, which is considered as a surrogate marker for protection^15,34^, and at least a twofold increase of titer from baseline (or titer above the highest level of measurement).

Secondary endpoints included the antibody levels as measured by NT after third vaccination and by ELISA after second and third vaccination, the assessment of immune reconstitution at baseline by measurement of immunoglobulin levels and analysis of lymphocyte subpopulations by flow cytometry, and the evaluation of safety data.

### Laboratory analyses

Serum samples were collected prospectively at baseline before first vaccination, 4 weeks after second, and 4 weeks after third vaccination. All serum samples were stored at −20 °C and analyzed by NT and ELISA. In addition, serum samples from patients and sibling donors, which were stored on average 1 month before transplantation during routine virological examinations, were retrospectively analyzed. All samples were labeled with consecutive numbers, and the laboratory staff was blinded and did not receive information concerning which blood samples were from patients and which from controls.

Neutralization assay is considered as gold standard and neutralizing antibodies contained in human serum are used as surrogate parameter for protection against TBE^15,34^. NTs were carried out by Pfizer reference laboratory situated in Orth, Austria. Serial dilutions of samples were incubated with approximately 100 tissue culture infective doses of TBEV for 2.5 h and replicates of mixtures were incubated for 7 days on TBEV-susceptible Vero cells seeded in 96-well microtiter plates^35^. Resulting supernatants were tested for the presence of TBEV by ELISA^36^. Microtiter plates were coated with 100 µl guinea pig anti-TBE immunoglobulin G (IgG) serum (Fa. Baxter AG) in a carbonate buffer. In all, 100 µl cell culture supernatants were added and incubated for 1 h at 36 °C. Plates were washed and 100 µl rabbit anti-TBE IgG serum (Fa. Baxter AG) were added and incubated for 1 h at 36 °C. After washing, 100 µl of a peroxidase-labeled donkey anti-rabbit IgG conjugate (Jackson Immuno Research Lab. Inc.) was added, incubated 1 h at 36 °C, and then washed again before the addition of 140 µl substrate (o-phenyldiamine in citrate phosphate buffer pH 5.0, plus 0.03% hydrogen peroxide). The enzymatic reaction was stopped after 15 min by the addition of 100 µl 0.25 M H_2_SO_4_, and color development was quantified by reading the optical density at 490 nm. The sample dilution resulting in virus neutralization in 50% of the replicates (NT_50_) was calculated using the method of Spearman and Karber. A cut-off value was set to 0.05 based on the titration of a known concentration of TBE viral antigen.

The Center for Virology of the Medical University of Vienna tested all samples by ELISA. TBE IgG ELISAs were carried out as previously described using non-treated microtiter plates coated with 0.5 µg/ml highly purified TBEV (strain Neudörfl) and tenfold dilutions of human sera, starting at 1:100^37,38^. For detection, biotin-labeled goat anti-human IgG (Pierce) and streptavidin-conjugated peroxidase (Sigma) were used. Specific IgG was quantified in arbitrary units (AU) with a standard polyclonal human anti-TBEV serum set at 1000 AU. Twofold serial dilution curves of the standard (seven data points) were fitted using a four-parameter logistic regression. The definition of the cut-off was based on the validation of the assay with 90 flavivirus-negative sera (positive ≥220 Vienna Units).

In addition, the status of immune reconstitution in HSCT recipients prior to vaccination was assessed from fresh whole blood using immunofluorescence staining and flow cytometric analyses (fluorescence-activated cell sorting (FACS)). The following cell populations were determined: leukocytes, granulocytes, monocytes, lymphocytes, T lymphocytes (CD3+), T helper cells (CD4+), naive and memory T helper cells (CD4+CD45RA+ and RO+), T suppressor cells (CD8+), naive and memory T suppressor cells (CD8+CD45RA+ and RO+), B lymphocytes (CD19+), and B cell subsets (CD19+CD21low immature B cells, CD19+CD21highCD38+ IgMhigh transitional B cells, CD19+CD10−CD27−CD21high naive B cells, CD19+CD27+IgD+ and CD19+CD27+IgD− non-class- and class-switched memory B cells, and plasmablasts). All FACS sequential gating strategies are shown in Supplementary Fig. 1. Serum levels of Igs (IgG, IgM, IgA) were quantified by nephelometry.

### Statistical analysis

The calculation of the sample size was performed using nQuery 6.1. The primary endpoint was the outcome of the NT against 4 weeks after the second vaccination. Fisher exact test was calculated to analyze the primary hypothesis on the difference in NT titer response between patients and controls. Furthermore, a multivariable logistic regression model was applied accounting for group as well as age, BMI, and gender as possible influence factors. To measure the agreement between the NT and ELISA response, Cohen’s Kappa and the corresponding 95% CIs were calculated. For titer values, the geometric mean (GMT) was calculated and the corresponding two-sided 95% CIs were constructed by back-transformation of the CI for the mean of the logarithmically transformed results. To investigate the difference in absolute titer values and geometric mean fold rises between time points and groups, Wilcoxon tests were performed. To investigate possible influence factors on NT titer response within patients only, we first calculated univariate logistic regression models for each possible influence factor. Owing to the small sample size, Firth’s correction was applied in logistic regression models. All impact factors with *p* values <0.1 in univariate models were then further investigated using a multivariable logistic regression model with backward selection. All two-sided *p* values <0.05 were considered as statistically significant. All analyses were performed using R, version 3.3.3 and SPSS, version 23.

## Supplementary information

Supplementary Information

## Data Availability

The data that support the findings of this study are available at Mendeley data (https://data.mendeley.com/datasets/g46jcnjmkc/1). The full trial protocol is available at clinicaltrials.gov (https://clinicaltrials.gov/ct2/show/NCT01991067).
