# Author Correction: Humoral immune response to tick-borne encephalitis vaccination in allogeneic blood and marrow graft recipients

**DOI:** 10.1038/s41541-020-00230-2

**Published:** 2020-08-31

**Authors:** Nicole Harrison, Katharina Grabmeier-Pfistershammer, Alexandra Graf, Ilse Schwarzinger, Judith H. Aberle, Karin Stiasny, Hildegard Greinix, Werner Rabitsch, Peter Kalhs, Michael Ramharter, Heinz Burgmann, Christina Forstner

**Affiliations:** 1 Division of Infectious Diseases and Tropical Medicine, Department of Medicine I, Medical University of Vienna, Vienna, Austria; 2 Division of Clinical and Experimental Immunology, Institute of Immunology, Center for Pathophysiology, Infectiology and Immunology, Medical University of Vienna, Vienna, Austria; 3 Section of Medical Statistics, Center for Medical Statistics, Informatics and Intelligent Systems, Medical University of Vienna, Vienna, Austria; 4 Department of Laboratory Medicine, Medical University of Vienna, Vienna, Austria; 5 Center for Virology, Medical University of Vienna, Vienna, Austria; 6 Division of Hematology, Department of Internal Medicine, Medical University of Graz, Graz, Austria; 7 Bone Marrow Transplantation Unit, Department of Medicine I, Medical University of Vienna, Vienna, Austria; 8 Department of Tropical Medicine, Bernhard Nocht Institute for Tropical Medicine & I. Department of Medicine, University Medical Center Hamburg-Eppendorf, Hamburg, Germany; 9 Institute of Infectious Diseases and Infection Control, Jena University Hospital, Jena, Germany

Correction to: *npj Vaccines* 10.1038/s41541-020-00215-1, published online 24 July 2020

In the original version of this Article, the link supplied in the Data availability section to the data supporting this study was broken. This has now been corrected in both the HTML and PDF versions of the Article.

