# Supplementary material for: Humoral immune response to tick-borne encephalitis vaccination in allogeneic blood and marrow graft recipients
Source: NPJ Vaccines. Author manuscript; Available in PMC 2020 Jul 28. (PMC7381595; doi:10.1038/s41541-020-00215-1)
Supplement: Supplementary Figure 1 [file EMS88042-supplement-Supplementary_Figure_1.pdf]

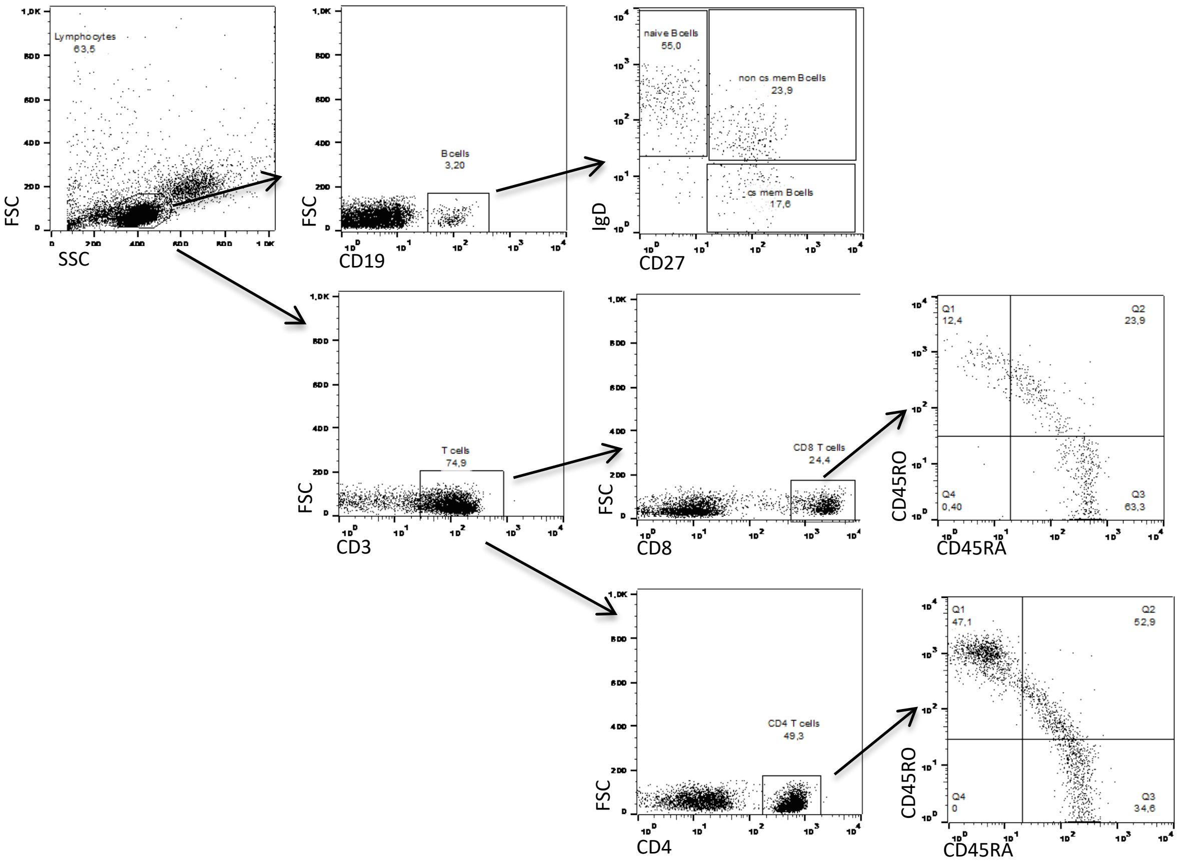

Supplementary Figure 1. FACS gating strategy
Lymphocytes were identified by FSC/SSC analysis from MNC (mononuclear cells). Within the lymphocyte gate D19 PerCP was used to identify B-cells. Memory B-cells were characterized as CD19 CD27 positive subset and further classified as class-switched or non-class-switched by IgD membrane expression. CD3 PerCP was used to identify T-cells within the lymphocyte gate. T-cells were further divided into CD8 and CD4 T-cells using APC labeled antibodies. CD4 and CD8 were further analysed based on their CD45RA and CD45RO expression.
